# Taxonomic Re-Examination of Nine *Rosellinia* Types (Ascomycota, Xylariales) Stored in the Saccardo Mycological Collection

**DOI:** 10.3390/microorganisms9030666

**Published:** 2021-03-23

**Authors:** Niccolò Forin, Alfredo Vizzini, Federico Fainelli, Enrico Ercole, Barbara Baldan

**Affiliations:** 1 Botanical Garden, University of Padova, Via Orto Botanico, 15, 35123 Padova, Italy; federico.fainelli@gmail.com; 2 Institute for Sustainable Plant Protection (IPSP-SS Torino), C.N.R., Viale P.A. Mattioli, 25, 10125 Torino, Italy; alfredo.vizzini@unito.it; 3 Department of Life Sciences and Systems Biology, University of Torino, Viale P.A. Mattioli, 25, 10125 Torino, Italy; ercole.enrico@gmail.com; 4 Department of Biology, University of Padova, Via Ugo Bassi, 58b, 35131 Padova, Italy

**Keywords:** fungal taxonomy, illumina sequencing, mycological collection, phylogeny, Pier Andrea Saccardo, Xylariaceae

## Abstract

In a recent monograph on the genus *Rosellinia*, type specimens worldwide were revised and re-classified using a morphological approach. Among them, some came from Pier Andrea Saccardo’s fungarium stored in the Herbarium of the Padova Botanical Garden. In this work, we taxonomically re-examine via a morphological and molecular approach nine different *Rosellinia* *sensu* Saccardo types. ITS1 and/or ITS2 sequences were successfully obtained applying Illumina MiSeq technology and phylogenetic analyses were carried out in order to elucidate their current taxonomic position. Only the ITS1 sequence was recovered for *Rosellinia areolata*, while for *R. geophila*, only the ITS2 sequence was recovered. We proposed here new combinations for *Rosellinia chordicola*, *R. geophila* and *R. horridula*, while for *R. ambigua*, *R. areolata*, *R. australis*, *R. romana* and *R. somala*, we did not suggest taxonomic changes compared to the current ones. The name *Rosellinia subsimilis* Sacc. is invalid, as it is a later homonym of *R. subsimilis* P. Karst. & Starbäck. Therefore, we introduced *Coniochaeta dakotensis* as a nomen novum for *R. subsimilis* Sacc. This is the first time that these types have been subjected to a molecular study. Our results demonstrate that old types are an important source of DNA sequence data for taxonomic re-examinations.

## 1. Introduction

The genus *Rosellinia* (Ascomycota, Xylariales), erected by De Notaris in 1844 [1] and typified by *Rosellinia aquila*, includes species that form superficial, dark brown to black, ostiolate stromata usually embedded in a mat of hyphae called subiculum, with each stroma containing one or sometimes few perithecia. Perithecia produce unitunicate, cylindrical asci with an amyloid apical ring (apparatus) and unicellular, asymmetrically ellipsoid, brown, often with a germ slit ascospores [2,3]. They occur in temperate and tropical regions as saprobes, some as endophytes and a few as root pathogens of economic important plant species (e.g., *Vitis vinifera*), growing mainly on deciduous woods of dicotyledonous plants [2]. *Rosellinia* species have a geniculosporium-like asexual morph and rarely a nodulisporium-like stages [3,4]. Over time, *Rosellinia* was synonymized with other genera (e.g., *Dematophora*) and placed in different families until Miller in 1928 [5] considered it as a genus of the family Xylariaceae, a classification now also confirmed by phylogenetic studies [4,6].

In 1882, Pier Andrea Saccardo [7] subdivided *Rosellinia* into different sections on the basis of stromatal features. *Eu-Rosellinia* with large, non-setose stromata immersed in a subiculum; *Calomastia* with large, non-setose stromata without subiculum; *Tassiella* with large, verrucose, non-setose stromata; *Amphisphaerella* (Cfr. *Anthostomella*) with the base of the stromata immersed; *Coniomela* with small, non-setose gregarious ascomata; *Coniochaeta* with small, setose, gregarious ascomata; *Cucurbitula* with erumpent ascomata; and *Sphaeropyxis* with short stipitate ascomata and globose ascospores. Moreover, he added the sections of lichenicolous species and doubtful species [2,3]. He described new *Rosellinia sensu* Saccardo species, many of them stored in his personal mycological collection at the Herbarium of the Padova Botanical Garden (PAD). In Saccardo’s fungarium, the genus includes 75 different species, with more or less 35 represented by type specimens [8]. Among Saccardo’s sections, *Coniochaeta* was raised to generic rank by Cooke in 1887 [9] and is currently accepted as genus of the family Coniochaetaceae (Sordariomycetes, Coniochaetales).

Recently, Petrini revised *Rosellinia* on the basis of the morphological characters of type specimens worldwide accepting in the genus 142 species and excluding 137 [3]. She subdivided the species in seven morphological groups within three different subgenera (*Rosellinia*, *Calomastia* and *Corrugata*) previously introduced by herself to accommodate species of the two Saccardo’s sections *Eu-Rosellinia* and *Calomastia* [2,3]. Species of *Rosellinia aquila*, *R. necatrix* and *R. buxi* groups were placed in the subgenus *Rosellinia*; species of *R. mammaeformis* and *R. mammoidea* groups in the subgenus *Calomastia*. The *Rosellinia emergens* group included species of both *Rosellinia* and *Calomastia* subgenera, while the species of the subgenus *Corrugata* were considered distinct from the other morphological groups and placed in the *R. thelena* group [3]. The multigene phylogeny study of Xylariales published by Wendt et al. [6] suggested that *Rosellinia sensu* Petrini [3] could be paraphyletic. Indeed, *R. necatrix* and *R. buxi* appeared as a sister clade of a clade containing the type species *R. aquila*. Wittstein et al. [4], through a multigene phylogeny and secondary metabolites study, excluded the species of the *Rosellinia necatrix* and *R. buxi* groups from *Rosellinia*. These species were accommodated in the resurrected genus *Dematophora*, previously considered a synonym of *Rosellinia* [4].

In this work, a molecular phylogenetic study, based on the nucleotide sequences of the internal transcribed spacer region (ITS) obtained by applying an Illumina MiSeq technology, was carried out with the aim of defining the current taxonomy of a sub-sample of more than 100-year-old *Rosellinia* type collections stored in Saccardo’s mycological collection. The molecular study was also coupled with new morphological observations of the type specimens.

## 2. Materials and Methods

### 2.1. Specimens Sampling and Morphology

Fungal specimens (indicated in bold in Table 1 and Table 2) were observed with a stereomicroscope Leica EZ4W to sample a small number of dried stromata/ascomata with sterilized tweezers. The material was used for both new morphological and molecular characterizations.

One or two stromata/ascomata were placed on a glass slide, rehydrated in water and smashed up. The characteristics of asci and ascospores, if present, were observed adding 3% lactic acid solution of Cotton Blue, while the presence of an amyloid ascal apical apparatus was tested pre-treating other stromata/ascomata with 10% potassium hydroxide (KOH) and then with Lugol’s solution. Ascospores and asci were observed using an optical microscope Leica DM500 with 400× or 1000× magnifications and photographed with a Leica ICC50W camera integrated in the optical microscope. Stromata/ascomata were photographed with a stereomicroscope Leica EZ4W. They resulted usually collapsed, so their shape and size were recorded when possible. All the measurements were taken using Fiji [10]. Measures of asci and ascospores are indicated as: (minimum–) average minus standard deviation—*average*—average plus standard deviation (–maximum) of length × (minimum–) average minus standard deviat—*average*—average plus standard deviation (–maximum) of width. In addition, spore quotient (Q; length/width ratio) = (minimum–) average minus standard deviat—*average*—average plus standard deviation (–maximum), and average spore quotient (Q_av_) are reported.

### 2.2. DNA Extraction, PCR Amplification, Sequencing and Data Analysis

DNA was extracted with the CTAB protocol described in Forin et al. [11]. In order to prepare the libraries for a paired-end sequencing using the Illumina MiSeq technology 2 × 300 bp, ITS1 and ITS2 regions were amplified using a two-step PCR process [12]. The ITS1 region was first amplify using the universal fungal primers ITS1f/ITS1 and ITS2 [13,14], while the ITS2 region with ITS3 and ITS4 [14]. The second PCR was carried out using the products of the first PCR and the same couple of primers used in the first one tagged with different 5 bp identifier tags. The tags are necessary to distinguish the sequences coming from each different type. The second PCR was done in four replicates for each couple of tagged primers. The first PCR was performed in a total volume of 25 μL containing 5 μL of 5X Wonder Taq reaction buffer (EuroClone; 5 mM dNTPs, 15 mM MgCl_2_), 0.5 μL of bovine serum albumin (BSA, 10 mg/mL), 0.5 μL each of two primers (10 μM), 0.5 μL of Wonder Taq (5 U/μL), 2 μL of genomic DNA and water to reach the final volume. The second PCR was performed without the BSA, using 2 μL of amplicons from the first PCR as template and the primers with tags. The PCR conditions were set as follows: initial denaturation at 95 °C for 3 min; 35 cycles consisting of a denaturation at 95 °C for 30 s, an annealing at 53 °C for ITS1 region and 54 °C for ITS2 region for 40 s and an extension at 72 °C for 45 s; and a final extension at 72 °C for 5 min. The PCRs were purified with the PureLink PCR Purification Kit (Thermo Fisher Scientific, Waltham, MA, United States) and quantified with Qubit dsDNA HS Assay Kit (Thermo Fisher Scientific, Waltham, MA, United States). Amplicons from different samples were mixed in equimolar amount to prepare ITS1 and ITS2 libraries, in accordance with the specifications provided by Fasteris sequencing service (Plan-les-Ouates, Switzerland).

Forward and reverse fastq files from each library were merged using PEAR v. 0.9.10 [15]. The merged reads were demultiplexed and quality filtered with QIIME v. 1.9.1 [16]. The parameters used are the same reported in Forin et al. [12]. VSEARCH v. 2.3.4 [17] was used to dereplicate the sequences, to filter out chimeric sequences and to cluster the sequences into Operational Taxonomic Units (OTUs). ITS1 and ITS2 regions were extracted using ITSx [18]. To perform the OTUs clustering a 98% similarity threshold was used. OTUs represented by fewer than 10 sequences were discarded, and the Fungi UNITE+INSD dataset v. 8.0 [19] for QIIME was used as reference for the taxonomic assignment. The OTUs were also compared with the sequences of the National Center for Biotechnology Information (NCBI) using a BLASTn search [20], excluding uncultured/environmental sample sequences. The final OTU abundance table was created with VSEARCH, considering an identity value of 98%.

### 2.3. Phylogenetic Analysis

The sequences used for the phylogenetic analyses are reported in Table 1 and Table 2. Three different datasets were generated according to the final taxonomic assignments and BLAST results: an ITS dataset of *Coniochaeta* species; an ITS dataset of taxa of the family Podosporaceae; and a combined dataset with ITS, 28S nuclear ribosomal RNA gene (LSU) and β-tubulin gene (*TUB2*) data of taxa of the families Xylariaceae and Hypoxylaceae. ITS1 and ITS2 sequences, when both identified, of the Saccardo types were combined and used in the phylogenetic analyses.

The sequences were aligned using the online version of MAFFT v. 7 [21] and manually refined with Geneious R11.1.5 (https://www.geneious.com, accessed date: 25 February 2021). Phylogenetic analyses were performed using Maximum likelihood (ML) with RAxML-NG v. 1.0.1 [22] and Bayesian Inference (BI) with MrBayes v. 3.2.6 [23] in the CIPRES science gateway [24]. The best-fit models were estimated by the Bayesian information criterion (BIC) using jModelTest 2 [25] to provide a substitution model for each single alignment. We used the Tamura-Nei model with gamma distribution (TrN + G) for the *Coniochaeta* ITS dataset and the transition model with gamma distribution (TIM2 + G) for the Podosporaceae ITS dataset. In the combined dataset, for ITS and *TUB2* we used the Hasegawa-Kishino-Yano model with proportion of invariable sites and gamma distribution (HKY + I + G) while, for LSU, the Tamura-Nei model with equal base frequencies and proportion of invariable sites (TrNef + I). ML analyses were performed with 1000 bootstrap replicates. BI analyses were performed with two independent Monte Carlo Markov Chains (MCMC) runs, each with four chains of 10 M generations. Trees were sampled every 1000 generations and the first 25% were discarded as burn-in. A majority rule consensus tree of the remaining 10001 trees was calculated to obtain estimates for Bayesian posterior probabilities (BPP). Significance threshold was set ≥0.95 for Bayesian posterior probability (BPP) and ≥70% for ML bootstrap values (MLB).

## 3. Results

### 3.1. Phylogenetic Analysis

**Figure 1:**
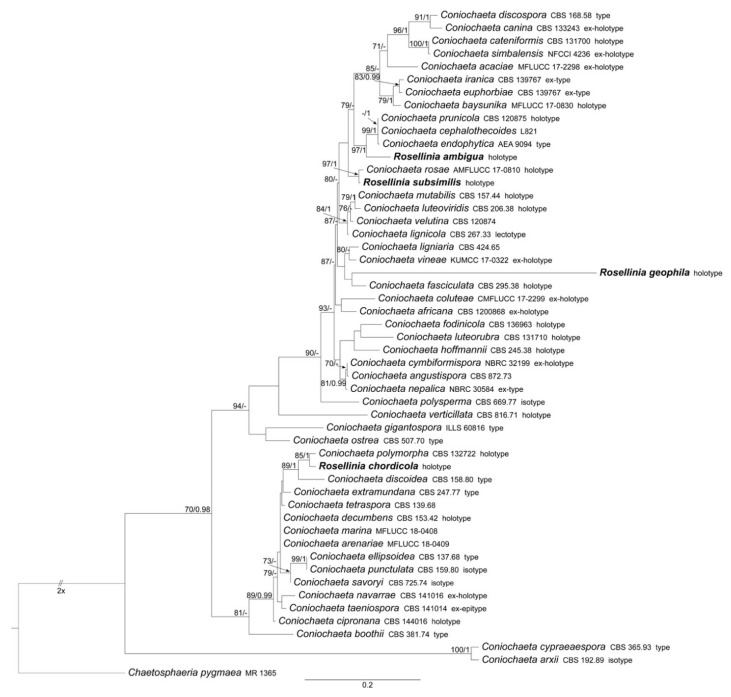
RAxML phylogram obtained from ITS sequences of selected *Coniochaeta* species. *ChaetoScheme 70*. (left) and BPP values ≥0.95 (right) are shown on the branches. Newly obtained sequences are reported in **bold**.

**Figure 2:**
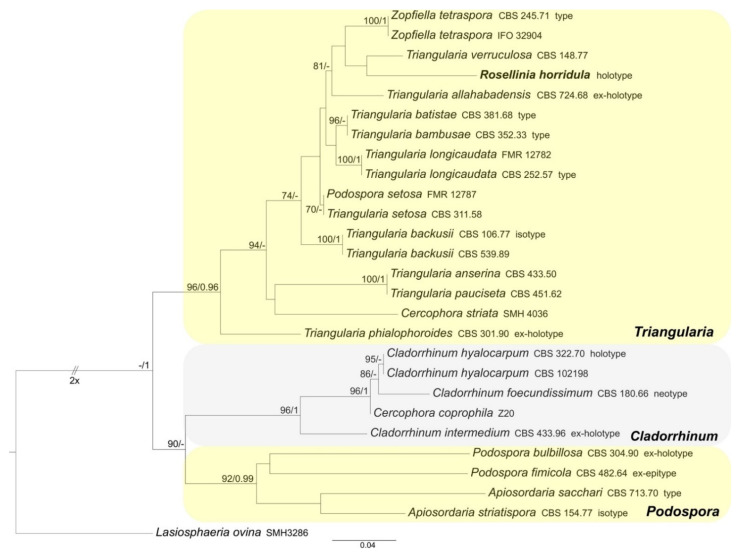
RAxML phylogram obtained from ITS sequences of selected *Triangularia*, *Cladorrhinum* and *Podospora* (Podosporaceae) species. *Lasiosphaeria ovina* (Lasiosphaeriaceae) was selected as the outgroup taxon. MLB values ≥70% (left) and BPP values ≥0.95 (right) are shown on the branches. Newly obtained sequence is reported in **bold**.

**Figure 3:**
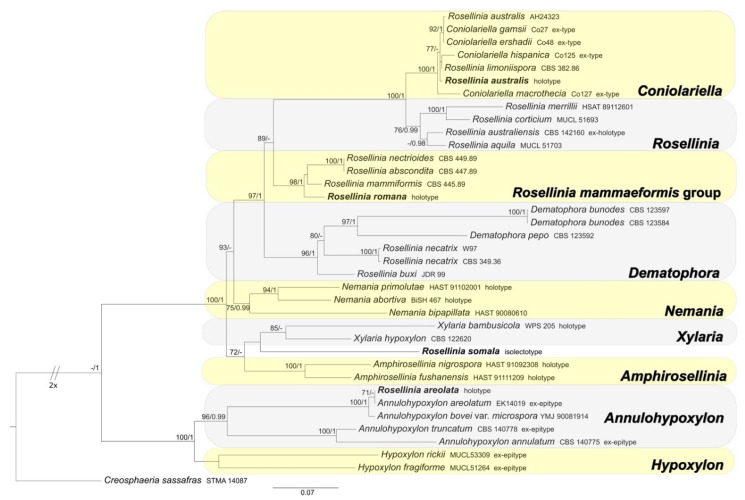
RAxML phylogram obtained from the combined ITS, LSU and *TUB2* sequences of selected species belonging to genera of Xylariaceae and Hypoxylaceae. *Creosphaeria sassafras* (Lopadostomataceae) was selected as the outgroup taxon. MLB values ≥70% (left) and BPP values ≥0.95 (right) are shown on the branches. Newly obtained sequences are reported in **bold**.

ML and BI analyses produced trees with congruent topologies. Therefore, the trees obtained from the RAxML-NG analysis with MLB and BPP values are reported (Figure 1, Figure 2 and Figure 3).

#### 3.1.1. Coniochaeta

The *Coniochaeta* dataset includes 52 ITS sequences: four newly generated; 47 *Coniochaeta* ITS sequences and *Chaetosphaeria pygmaea* (the outgroup, following Wanasinghe et al. [34]) obtained from GenBank (Table 1). The alignment comprises 563 characters, including indels and missing data. *Rosellinia ambigua*, *R. chordicola*, *R. geophila* and *R. subsimilis* fall within this genus (Figure 1). The discussion about these *Rosellinia* types is reported in the taxonomy section.

#### 3.1.2. Podosporaceae

The Podosporaceae dataset includes 27 ITS sequences: one newly generated; 26 Podosporaceae ITS sequences and *Lasiosphaeria ovina* (the outgroup, following Marin-Felix et al. [27]) obtained from GenBank (Table 1). The alignment comprises 558 characters, including indels and missing data. *Triangularia*, *Cladorrhinum* and *Podospora* form three distinct and well-supported clades (MLB = 96%, BPP = 0.96; MLB = 96%, BPP = 1 and MLB = 92%, BPP = 0.99, respectively). *Rosellinia horridula* is nested in the *Triangularia* clade (Figure 2).

#### 3.1.3. Xylariaceae and Hypoxylaceae

The Xylariaceae and Hypoxylaceae combined dataset includes 37 ITS sequences (4 newly generated, 33 obtained from GenBank); 20 28S (LSU) sequences (all obtained from GenBank); 22 *TUB2* sequences (all obtained from GenBank). *Creosphaeria sassafras* was selected as outgroup taxon following Wendt et al. [6]. The alignment comprises 712 (ITS) + 761 (LSU) + 1713 (*TUB2*) characters, respectively, with a total of 3186 characters, including indels and missing data. *Rosellinia australis* results included in the *Coniolariella* clade (MLB = 100%, BPP = 1); *R. romana* in the *Rosellinia mammaeformis sensu* Petrini clade (MLB = 98%, BPP = 1), while *R. areolata* clusters with *Annulohypoxylon areolatum* sequences in a highly supported clade (MLB = 100%, BPP = 1) (Figure 3). *Rosellinia somala* occupies an isolate position in the phylogram, sister to the *Xylaria* clade (Figure 3).

### 3.2. Taxonomy

#### 3.2.1. *Rosellinia ambigua*

***Coniochaeta ambigua*** (Sacc.) Popushoi, Mikoflora plodovykh derevyaev SSSR [Mycoflora of fruit trees of the U.S.S.R.] (Moscow): 90. 1971.

*Basionym*: *Rosellinia ambigua* Sacc., Atti Soc. Veneto-Trent. Sci. Nat. 2: 328. 1882.

*Sexual stage: Ascomata* perithecial, superficial, solitary to gregarious, black, globose with black setae on the surface (41–59 × 4.5–6 μm), papillate, 165–220 µm diam (*n* = 10); peridium not cephalothecoid. *Asci* cylindrical without amyloid apical apparatus, 90 × 9.6 μm (*n* = 1), 8-spored, ascospores obliquely uniseriate. *Ascospores* broadly-ellipsoidal, with round ends and a convex side giving them also a reniform shape, (7.7–)9.4–*10.5*–11.7(–13.3) × (5.5–)6.2–*7.1*–8(–8.8) μm, Q = (1–)1.3–*1.5*–1.7(–2), Q_av_ = 1.5 (*n* = 37), hyaline to yellow and brown at maturity, smooth, one-celled, with a straight germ slit as long as the ascospore, one-celled.

**Figure 4:**
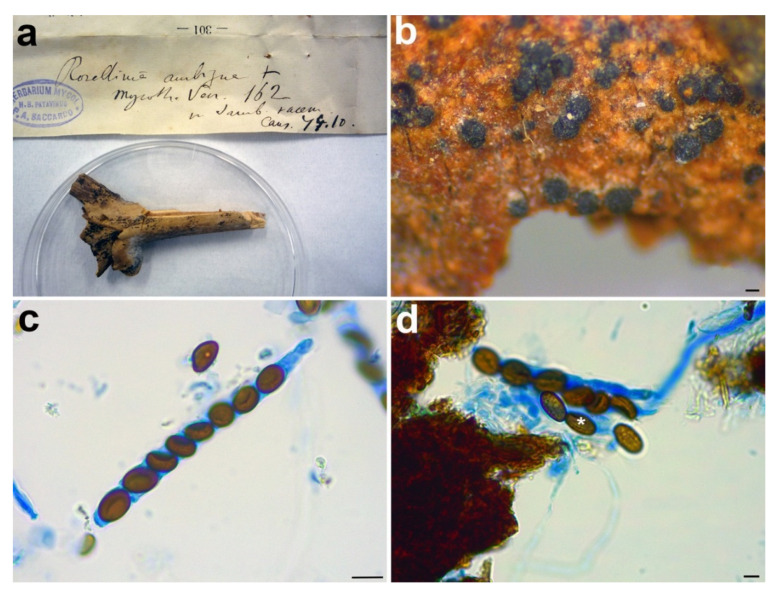
*Rosellinia ambigua*. (**a**) Original fungarium specimen. (**b**) Perithecia on natural substrate. (**c**) Ascus with ascospores. (**d**) Ascospores showing germ slit (white asterisk). Scale bars: (**b**) = 100 μm; (**c**) = 10 μm; (**d**) = 5 μm.

*Material examined:* ITALY, Cansiglio, on *Sambucus racemosa*, ? October 1879, n. 162, PAD S00027, holotype (Figure 4).

*Notes: Rosellinia ambigua* was moved to the genus *Coniochaeta* (asexual morph *Lecythophora*) by Popushoi in 1971 [60]. The genus is characterized by species with perithecial, pyriform to globose ascomata, cylindrical asci without amyloid apical apparatus and ellipsoid to globose, one-celled and brown ascospores often laterally compressed with a germ slit [31,46]. The morphological observation of *Rosellinia ambigua* fits well with the general phenotypic traits of *Coniochaeta* reported above (e.g., brown and globose ascospores with a germ slit). In the *Coniochaeta* phylogram (Figure 1), *Rosellinia ambigua* clusters in a highly supported clade (MLB = 97%, BPP = 1) where it is sister to a clade (MLB = 99%, BPP = 1) consisting of the ITS sequences of *C. cephalothecoides*, *C. prunicola* and *C. endophytica*. The molecular analysis of the holotype confirms the taxonomic re-classification proposed by Popushoi [60]. *Coniochaeta endophytica* was described only from its asexual stage [39]. *Coniochaeta prunicola* has ascospores similar to *Rosellinia ambigua* (9.2 ± 0.6 × 6.7 ± 0.6 μm) but it differs in the dimension of ascomata (200–250 μm diam), the presence of a long neck above the perithecia and shorter asci (av. 69 × 9.5 μm) [32]. *Coniochaeta cephalothecoides* and *Rosellinia ambigua* are morphologically very similar but the former shows a cephalothecoid peridium [61].

#### 3.2.2. *Rosellinia areolata*

***Annulohypoxylon areolatum*** (Sacc.) Sir & Kuhnert, in Kuhnert, Sir, Lambert, Hyde, Hladki, Romero, Rohde & Stadler, Fungal Diversity 85: 18. 2016.

*Basionym*: *Rosellinia areolata* Sacc., Ann. Mycol. 11: 314. 1913.

*Synonyms*: *Annulohypoxylon bovei* var. *microsporum* (J.H. Mill.) Y.M. Ju, J.D. Rogers & H.M. Hsieh (as *‘microspora’*), Mycologia 97: 857. 2005.

*Hypoxylon bovei* var. *microsporum* J.H. Miller, Monogr. of the World Species of Hypoxylon, p. 95. 1961.

*Hypoxylon marginatum* var. *mammiforme* Rehm, Leafl. Philipp. Bot. 8: 2958. 1916.

*Hypoxylon chalybeum* var. *effusum* Sacc. apud Sacc. & Trott., Syll. Fung. XXIV, p. 1080. 1928.

*Sexual stage*: *Stromata* gregarious, superficial, brown, spherical to spherical compressed, 0.9–1 mm diam (*n* = 8). Ostioles papillate, with annular disk, 0.4–0.5 mm diam. *Asci* not found. *Ascospores* asymmetrically ellipsoidal, with round ends and a convex side, (9.3–)9.6–*10.3*–10.9(–12) × (3.9–)4.2–*4.4*–4.7(–5.4) μm, Q = (2–)2.1–*2.3*–2.5(–2.8), Q_av_ = 2.3 (*n* = 40), hyaline and brown at maturity, smooth, with a straight germ slit spore-length, one-celled.

**Figure 5:**
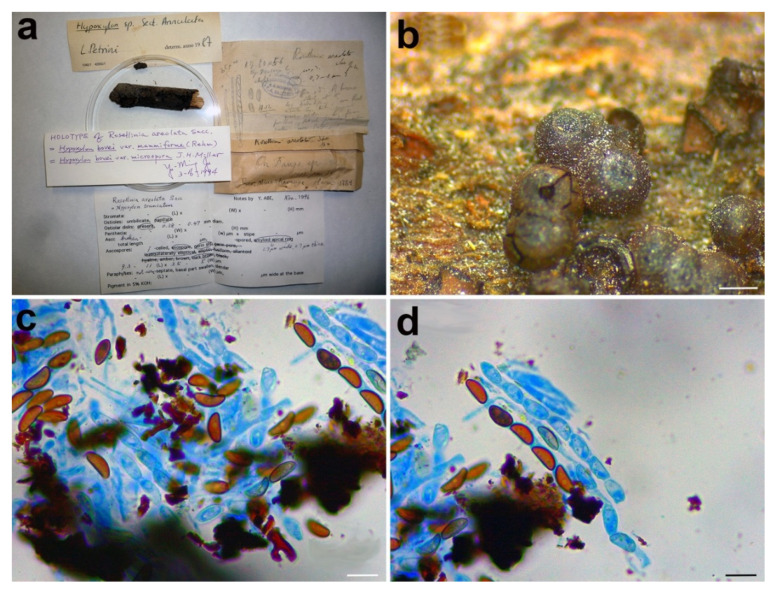
*Rosellinia areolata*. (**a**) Original fungarium specimen. (**b**) Perithecia on natural substrate. (**c**,**d**) Ascospores. Scale bars: (**b**) = 500 μm; (**c**,**d**) = 10 μm.

*Material examined*: JAPAN, Mino prov., Kawauye-mura (currently Gifu pref., Nakatsugawa city), on *Fagus* sp., 30 January 1913, K. Hara, PAD S00028, holotype (Figure 5).

*Notes*: In 1987, Petrini revised the holotype *Rosellinia areolata* considering it as a member of the genus *Hypoxylon* sect. *Annulata* (Figure 5a). In the monograph about *Rosellinia*, Petrini revised this species placing it in synonymy with *Annulohypoxylon bovei* var. *microsporum* [3]. In 1994 Yu-Ming Ju suggested a synonymy with *Hypoxylon bovei* var. *mammiforme* and *H. bovei* var. *microsporum* (basionym of *Annulohypoxylon bovei* var. *microsporum*). *Hypoxylon bovei* var. *mammiforme* does not seem to exist as nomenclatural name and we are convinced that the author intended *H. marginatum* var. *mammiforme* (= *Annulohypoxylon bovei* var. *microsporum*) (Figure 5a). In 1996, Abe reported a possible synonymy with *Hypoxylon truncatum*, today *Annulohypoxylon truncatum*, the type species of the genus *Annulohypoxylon*. *Annulohypoxylon areolatum* was recently proposed as new combination for *Rosellinia areolata* and *A. bovei* var. *microsporum* is treated as its synonym [49]. An epitype of *Annulohypoxylon areolatum* was designated in Kuhnert et al. [49]. The morphological observations of *Rosellinia areolata* fit with the description of *Annulohypoxylon areolatum* reported by Kuhnert et al. [49], which was not based on the holotype of *R. areolata*. *Annulohypoxylon* species are characterized by the presence of carbonaceous stromata enclosing perithecia, conic-papillate ostioles encircled with an annulate disk, and ascospore perispores with a thickened area visible in 10% KOH at circa 1⁄3 ascospore length when dehiscing [50]. The ITS1 sequence recovered from the holotype clusters with *Annulohypoxylon areolatum* (MFLUCC 14-1233, ex-epitype) and *A. bovei* var. *microspora* (YMJ 90081914) ITS sequences in a highly supported clade (MLB = 100%, BPP = 1) (Figure 3). The ITS1 sequences of *Rosellinia australis* and *Annulohypoxylon areolatum* have a nucleotide identity of 99.3%. The molecular analysis confirms the taxonomic reclassification proposed by Kuhnert et al. [49], excluding the synonymy with *Annulohypoxylon truncatum* suggested by Abe in the label (Figure 5a).

#### 3.2.3. *Rosellinia australis*

***Coniolariella limoniispora*** (Ellis & Everth.) Checa, Arenal & J.D. Rogers, Mycological Research 112: 797. 2008.

*Basionym*: *Rosellinia limoniispora* Ellis & Everh., Proc. Acad. Nat. Sci. Phila. 46:326. 1894.

*Synonyms*: *Coniolariella limonispora* var. *australis* Checa, Arenal & J.D. Rogers (as *‘limoniispora’*), Mycol. Res. 112: 797. 2008.

*Rosellinia australis* Sacc. & Trotter, Ann. Mycol. 11: 416. 1913, Nom. illegit., Art. 53.1, preoccupied by *Rosellinia australis* Speg., Anal. Mus. nac. B. Aires, Ser. 3, 12: 337. 1909. = *Rosellinia bonaerensis* Speg., Anal. Mus. nac. Hist. nat. B. Aires 6: 258. 1898. fide Petrini 2013.

*Sexual stage*: *Stromata* solitary to gregarious, superficial, black with the ostiolar region slightly papillate, globose, 582–904 µm diam (*n* = 10). *Asci* cylindrical without amyloid apical apparatus, (113–)113.7–*119.4*–125.2(–125.4) × (10.5–)10.6–*12.1*–13.6(–14) μm (*n* = 5), 8-spored, ascospores obliquely uniseriate. *Ascospores* citriform with apiculate ends, (13.3–)15.9–*17.5*–19.2(–20.8) × (7.6–)8.3–*9.1*–9.9(–10.8) μm, Q = (1.5–)1.7–*1.9*–2.2(–2.5), Q_av_ = 1.9 (*n* = 41), dark brown, smooth, with straight and long germ slit, one-celled, monoguttulate.

**Figure 6:**
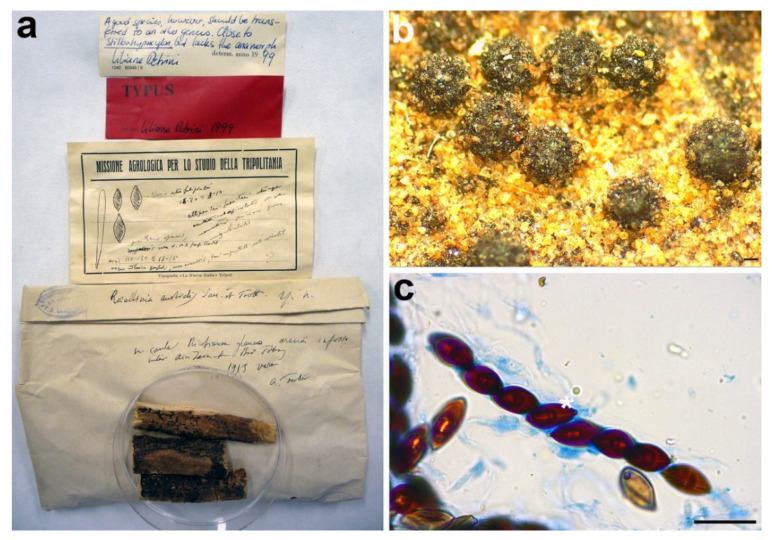
*Rosellinia australis*. (**a**) Original fungarium specimen. (**b**) Perithecia on natural substrate. (**c**) Ascus with ascospores; ascospore showing germ slit (white asterisk). Scale bars: (**b**) = 200 μm; (**c**) = 20 μm.

*Material examined:* LIBYA, Tripoli, on *Nicotiana glauca*, 1913, A. Trotter, PAD S00029, holotype (Figure 6).

*Notes*: The holotype was morphologically revised by Petrini in 1999, who excluded *Rosellinia australis* from *Rosellinia* (Figure 6a) due to the presence of soft stromata, perithecia adhering to the stromatal wall, asci without an amyloid apex and lack of a subiculum [57]. This species is now considered a synonym of *Coniolariella limoniispora* [51]. *Coniolariella* was introduced by García et al. [62] to accommodate the single species *Coniolariella gamsii*, previous placed in the genus *Coniochaeta* as *Coniochaeta gamsii,* characterized by stromata solitary or in small groups, globose and dark brown, asci cylindrical, without apical structures and ascospores one-celled, brown, ellipsoidal to citriform, with apiculate ends and a longitudinal germ slit. In a following molecular study, Checa et al. [57] added to *Coniolariella* the new species *C. hispanica* and proposed the new combination *C. limoniispora* for *Rosellinia limoniispora*. They recognized *Coniolariella gamsii*, the type species of the genus, and *Rosellinia australis* as varieties of *C. limoniispora*. Zare et al. [51] introduced the new species *Coniolariella macrothecia* and the new combination *Coniolariella ershadii* for *Coniochaeta ershadii*. In addition, they did not consider *Coniolariella gamsii* as a variety of *C. limoniispora* and synonymized *C. limoniispora* var. *australis* under *C. limoniispora*. In the genus, five different species are recognized [51]. The molecular study places the holotype *R. australis* in the highly supported *Coniolariella* clade (MLB = 100%, BPP = 1). Nevertheless, the use of two molecular markers (ITS and LSU, see Table 2) is not sufficient to delimit all the different species. The morphology of *Rosellinia australis* fits with the morphological description of *Coniolariella limoniispora* reported by Checa et al. [57], confirming that *Rosellinia australis* can be considered a synonym of *Coniolariella limoniispora*.

#### 3.2.4. *Rosellinia chordicola*

***Coniochaeta chordicola*** (Sacc.) Forin, Fainelli & Vizzini **comb. nov.** MycoBank MB838853.

*Basionym*: *Rosellinia chordicola* Sacc., Michelia 1: 372. 1878.

*Sexual stage*: *Ascomata* perithecial, solitary, superficial, black, globose, about 290 µm diam. *Asci* immature without amyloid apical apparatus. *Ascospores* broadly-ellipsoidal, with round ends and a convex side giving them also a reniform shape, (8.9–)10.1–*11.1*–12.1(–14.4) × (6.3–)7.5–*8.5*–9.6(–10.8) μm, Q = (1.1–)1.2–*1.3*–1.5(–1.6), Q_av_ = 1.3 (*n* = 46), brown, smooth with a straight germ slit nearly as long as the ascospore, one-celled.

**Figure 7:**
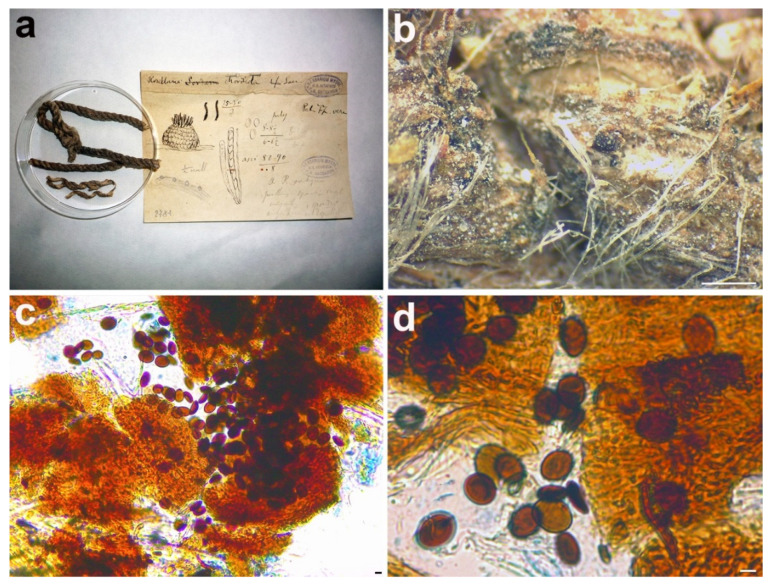
*Rosellinia chordicola*. (**a**) Original fungarium specimen. (**b**) Perithecia on natural substrate. (**c**,**d**) Ascospores. Scale bars: (**b**) = 500 μm; (**c**,**d**) = 5 μm.

*Material examined:* ITALY, Padova, Botanical Garden, on a rope, 1877, PAD S00030, holotype (Figure 7).

*Notes:* Phylogenetically, *Rosellinia chordicola* in the *Coniochaeta* genus is closely related to *C. polymorpha* (CBS 132722, holotype) and *C. discoidea* (CBS 158.80, type) (Figure 1). *Coniochaeta polymorpha* was morphologically described only based on its asexual stage [63]; therefore, a comparison between the sexual stages of Saccardo’s type and *C. polymorpha* was not possible. The ITS sequences of *Rosellinia chordicola* and *C. polymorpha* show an identity of 97% with 10 nucleotide differences. *Rosellinia chordicola* and *Coniochaeta discoidea* have discoid ascospores with similar dimensions but they differ in the ornamentation of the ascospores. *Coniochaeta discoidea* has ascospores (8–)9–12 × 8–11 μm characterized by the presence of circular to elongate pits [64]. Our molecular analysis suggests that *Rosellinia chordicola* should be treated as a distinct species within the genus *Coniochaeta*.

#### 3.2.5. *Rosellinia geophila*

***Coniochaeta geophila*** (E. Bommer, M. Rousseau & Sacc.) Forin, Fainelli & Vizzini **comb. nov.** MycoBank MB838854.

*Basionym*: *Rosellinia geophila* E. Bommer, M. Rousseau & Sacc., in Saccardo, Ann. Mycol. 3: 508. 1906.

*Sexual stage*: *Ascomata* perithecial, solitary or gregarious, superficial, black, globose with black setae on the surface (66.4–101.2 × 6–8.9 μm) and a slightly papillate ostiolar region, 349–448 µm diam (*n* = 5). *Asci* not found. *Ascospores* ellipsoidal with broadly rounded ends, (18.9–)23–*26*–29.1(–30.7) × (9.9–)11–*12.6*–14.2(–16) μm, Q = (1.4–)1.8–*2.1*–2.4(–2.7), Q_av_ = 2.1 (*n* = 50), brown, smooth, with a straight germ slit as long as the ascospore, one-celled.

**Figure 8:**
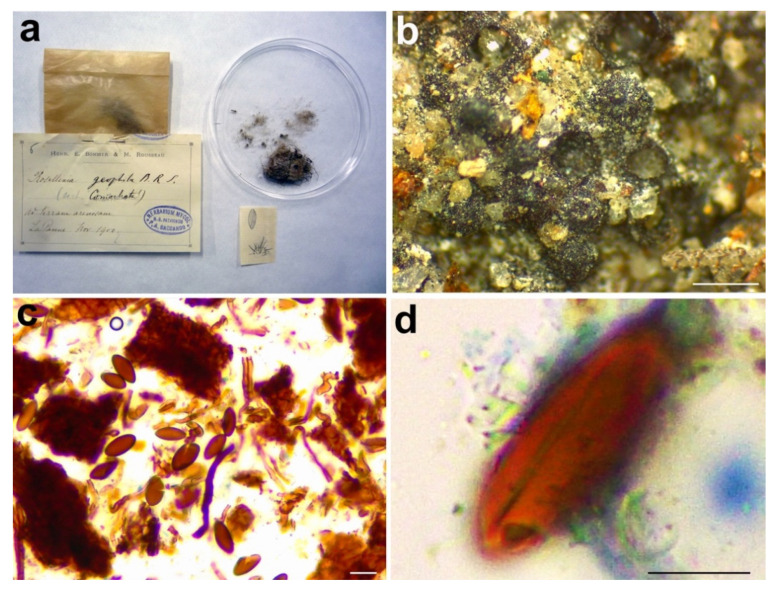
*Rosellinia geophila*. (**a**) Original fungarium specimen. (**b**) Perithecia on natural substrate. (**c**) Ascospores. (**d**) Ascospore showing germ slit. Scale bars: (**b**) = 500 μm; (**c**) = 20 μm; (**d**) = 10 μm.

*Material examined*: BELGIUM, La Panne pr. Furnes, on sandy ground among mosses, November 1900, PAD S00031, holotype (Figure 8).

*Notes*: Only the ITS2 sequence has been obtained from *Rosellinia geophila*. The molecular analysis places the holotype close to *Coniochaeta fasciculata* (CBS 295.38, holotype), only known for its asexual morph [65]. *Rosellinia geophila* clusters also with *Coniochaeta lignaria* and *C. vineae* (Figure 1). *C. vineae* has ascomata covered by setae and brown ascospores with a straight germ slit, but it differs from *R. geophila* in having smaller ascomata (170–185 μm diam) and smaller ascospores (6.5–9.5 × 4–6 μm) ovoidal and multi-guttulate [46]. *Coniochaeta ligniaria* has pointed setae often covering the whole ascomata and brown ellipsoidal ascospores with a germ slit smaller than those of *Rosellinia geophila* (12–15 × 8–10 μm) [66,67]. The results of the taxonomic assignment coupled with the phylogenetic analysis (Figure 1) suggest that this is a distinct *Coniochaeta* species confirming the original placement of *Rosellinia geophila* in the genus *Rosellinia* sect. *Coniochaeta* as reported in the original label (Figure 8a).

#### 3.2.6. *Rosellinia horridula*

***Triangularia horridula*** (Sacc.) Forin, Fainelli & Vizzini **comb. nov.** MycoBank MB838855.

*Basionym*: *Rosellinia horridula* Sacc., Fl. Sard. Comp.: 248. 1884.

*Synonym*: *Podospora horridula* (Sacc.) Dennis & S.M. Francis, Trans. Br. Mycol. Soc. 82: 380. 1984.

*Sexual stage*: *Ascomata* solitary or gregarious, pyriform to ovate with a short conical neck, superficial or immersed with only the neck protruding, black, covered with flexuous hairs, about 400 µm diam. *Asci* not found. *Ascospores* ellipsoidal with an apical end rounded while the other one flattened, inequilateral and slightly curved, (26.2–)29–*32.5*–36(–41.8) × (12.5–)13.5–*15.1*–16.7(–17.6) μm, Q = (1.7–)1.9–*2.2*–2.4(–2.5), Q_av_ = 2.2 (*n* = 15), dark brown, smooth, one-celled.

**Figure 9:**
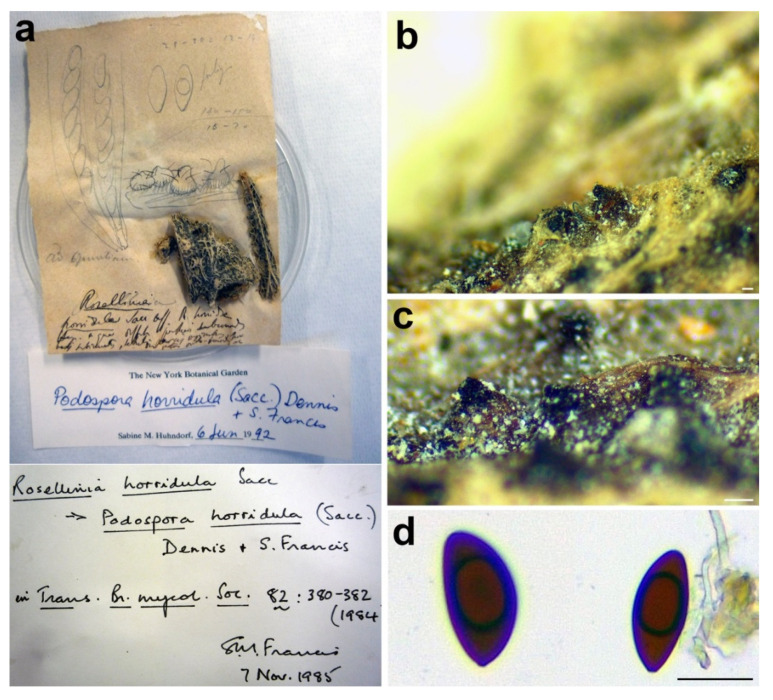
*Rosellinia horridula*. (**a**) Original fungarium specimen. (**b**,**c**) Perithecia on natural substrate. (**d**) Ascospores. Scale bars: (**b**,**c**) = 100 μm; (**d**) = 20 μm.

*Material examined*: ITALY, Sardinia, Torralba, on *Opuntiae* sp., Marcucci, PAD S00032, holotype (Figure 9).

*Notes*: The specimen was morphologically revised by S.M. Francis in 1985 and by S.M. Huhndorf in 1992, as reported in the labels associated with the sample (Figure 9a). *Rosellinia horridula* was redescribed from the holotype as *Podospora horridula* [68]. Wang et al. [30] introduced the new family Podosporaceae to accommodate three different genera (*Podospora*, *Cladorrhinum* and *Triangularia*) forming a phylogenetic sister lineage of Chaetomiaceae in the Sordariales. *Podospora*, *Cladorrhinum* and *Triangularia* were re-defined, and many species previously identified as *Podospora*, including the genetic model species *P. anserina*, were moved to *Triangularia* [30]. In *Podospora sensu stricto*, only the type species *P. fimicola* was maintained and the new combination *P. bulbillosa* was proposed for *Cladorrhinum bulbillosum* [30]. Marin-Felix et al. [27] introduced as new combinations the species *Podospora striatispora* (= *Apiosordaria striatispora*), *P. costaricensis* (= *Cercophora costaricensis*) and *P. sacchari* (= *Apiosordaria sacchari*) in *Podospora sensu stricto* based on a phylogenetic study. The new genera *Rhypophila* (Naviculisporaceae) and *Pseudoechria* (Schizotheciaceae) were erected to accommodate different *Podospora* species. *Podospora cochleariformis*, *P. decipiens*, *P. myriaspora* and *P. pleiospora* in *Rhypophila*. *Podospora curvicolla*, *P. longicollis*, *P. decidua* and *P. prolifica* in *Pseudoechria* [27]. Our molecular analysis places *Rosellinia horridula* in the clade *Triangularia* close to *Triangularia verruculosa* (Figure 2), from which it differs for the presence of longer and one-celled ascospores (*T. verruculosa* has (23–)25.5–28.5(–29.5) μm long and two-celled ascospores) [30]. Therefore, a new combination is proposed here for *Rosellinia horridula*. *Triangularia* was restricted to the type species of the genus, *T. bambusae*, together with species characterized by two-celled ascospores that occurred in the same monophyletic clade [30]. Subsequently, a species with one-celled ascospores (*Arnium arizonense*) has been moved in *Triangularia* incorporating in the description of the genus also species with one-celled ascospores [27]. *Rosellinia horridula* has one-celled ascospores and represents the second species with this trait in the genus *Triangularia*.

#### 3.2.7. *Rosellinia romana*

***Rosellinia glabra*** (Fuckel) L.E. Petrini, Sydowia 44: 243. 1992.

*Basionym*: *Rosellinia aquila* var. *glabra* Fuckel, Symb. Myc. 149. 1869.

*Synonym*: *Rosellinia romana* Sacc., Annales Mycologici 10: 316. 1912.

*Sexual stage*: *Stromata* gregarious in small groups, superficial, black, globose, slightly papillate, 675–910 µm diam (*n* = 10). *Asci* cylindrical with amyloid apical apparatus, (92.8–)94.4–*102.6*–110.8(–112.8) × (8.7–)8.9–*9.6*–10.3(–10.8) μm (*n* = 6), 8-spored, ascospores obliquely uniseriate. *Ascospores* ellipsoidal to asymmetrical ellipsoidal with pinched ends, (10.4–)12.2–*13.1*–14(–14.5) × (4.6–)5.4–*5.9*–6.3(–7.1) μm, Q = (1.7–)2–*2.3*–2.5(–3), Q_av_ = 2.2 (*n* = 48), dark brown, smooth with a straight germ slit nearly as long as the ascospore, one-celled.

**Figure 10:**
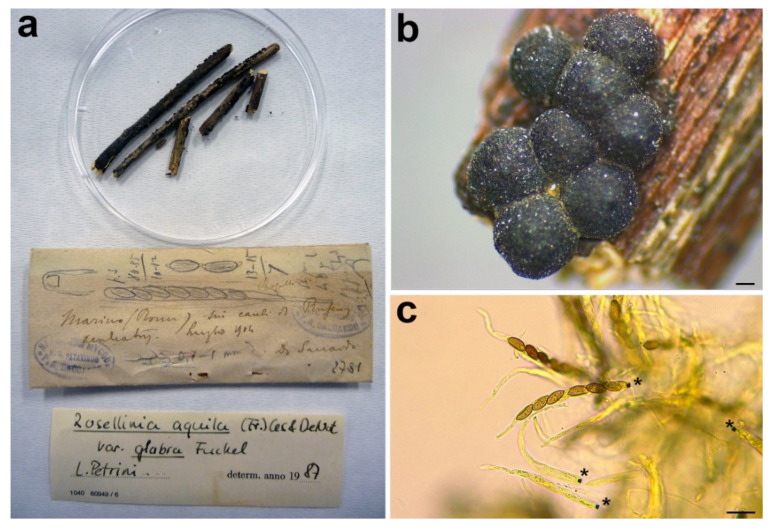
*Rosellinia romana*. (**a**) Original fungarium specimen. (**b**) Perithecia on natural substrate. (**c**) Asci with ascospores; black asterisks indicate the amyloid apical apparatus. Scale bars: (**b**) = 200 μm; (**c**) = 20 μm.

*Material examined*: Italy, Rome, Marino, on *Ruscus aculeatus*, July 1904, D. Saccardo, PAD S00033, holotype (Figure 10).

*Notes*: *Rosellinia romana* was morphologically revised by Petrini in 1987, as reported in the label associated with the specimen (Figure 10a), suggesting a synonymy with *R. aquila* var. *glabra*. In 1992 Petrini introduced the new combination *Rosellinia glabra* for *R. aquila* var. *glabra* and, as a consequence, *R. romana* became a synonym of *R. glabra* [2,3]. *Rosellinia glabra* is characterized by large stromata (0.7–0.9 mm diam), semiglobose to cupulate, brown, papillate, gregarious, forming small groups; ascospores (12.5) 16±2 (21) × (5.5) 6.5±0.5 (7.7) μm, ellipsoidal to asymmetrically ellipsoidal with broadly rounded or pinched ends, brown with a germ slit nearly as long as the ascospore, straight and a cellular appendage at one or both spore ends [2,3]. The ITS sequence of *R. romana* clusters in a highly supported clade (MLB = 98%, BPP = 1) with ITS sequences of *Rosellinia* species belonging to the *R. mammaeformis* group introduced by Petrini [3]. This morphological group should include also *Rosellinia glabra* [3]. However, a molecular comparison between *R. romana* and *R. glabra* was not possible because, for the latter, no molecular information is deposited in public databases. As already observed by Petrini [2], the morphologies of *R. romana* and *R. glabra* are very similar. In the absence of a molecular confirmation, we agree with the synonymy proposed by Petrini [2,3].

#### 3.2.8. *Rosellinia somala*

***Helicogermslita celastri*** (S.B. Kale & S.V.S. Kale) Lodha & D. Hawksw., Transactions of the British Mycological Society 81: 91. 1983.

*Synonym*: *Rosellinia somala* Bacc., Risultati scientifici della Missione Stefanini Paoli nella Somalia meridionale (Firenze): 195. 1916.

*Sexual stage*: *Stromata* solitary or in small groups of two/three, erumpent from bark and eventually almost superficial, black, globose, papillate, 572–793 µm diam (*n* = 5). *Asci* cylindrical without amyloid apical apparatus, 140.7–149.5 × 10.2–13.5 μm (*n* = 2), 8-spored, ascospores obliquely uniseriate. *Ascospores* ellipsoidal with rounded ends, (14.3–)15.8–*17.4*–19(–21.7) × (6–)7–*7.5*–8(–8.7) μm, Q = (1.8–)2.1–*2.3*–2.6(–3), Q_av_ = 2.3 (*n* = 48), dark brown, smooth, one-celled, with a helicoid germ slit coiling three times along the entire length of the ascospore.

**Figure 11:**
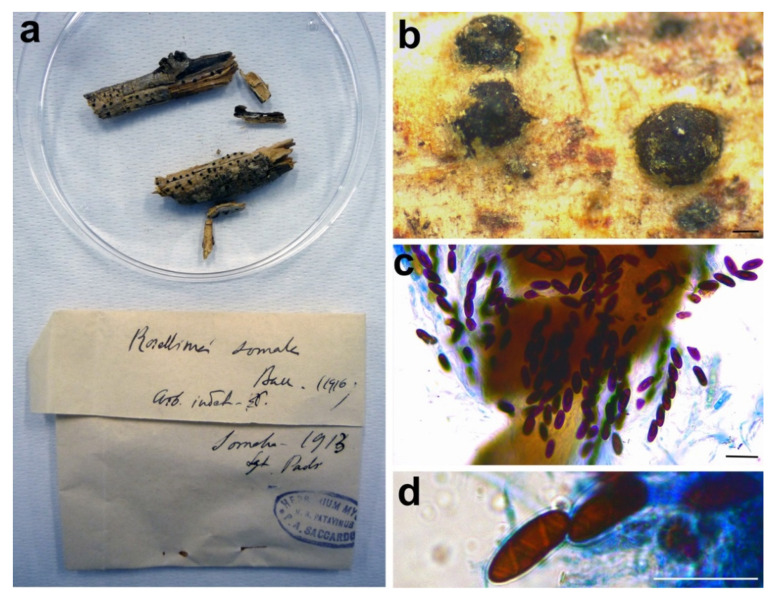
*Rosellinia somala*. (**a**) Original fungarium specimen. (**b**) Perithecia on natural substrate. (**c**) Ascospores. (**d**) Ascospore showing helicoid germ slit. Bars: (**b**) = 200 μm; (**c**,**d**) = 20 μm.

*Material examined*: SOMALIA, on branch, 1913, G. Paoli, PAD S00034, isolectotype (Figure 11).

*Notes*: Petrini [3] synonymized *Rosellinia somala* with *Helicogermslita celastri* after a morphological examination of the lectotype of *R. somala* stored in Harvard University (FH). *Helicogermslita* (Xylariaceae) was originally introduced to accommodate the single species *Helicogermslita celastri* (formerly *Amphisphaerella celastri*) [69]. Laessöe and Spooner [70] included in this genus other three previously described species: *Helicogermslita fleischhakii* (= *Sordaria fleischhakii*), *H. gaudefroyi* (= *Rosellinia gaudefroyi*), *H. valdiviensis* (= *Rosellinia valdiviensis*). Petrini [71] introduced three new species (*Helicogermslita gisbornia*, *H. johnstonii*, *H. mackenziei*) and the new combination *H. aucklandica* for *Rosellinia aucklandica*. Lee and Crous [72] described the new species *Helicogermslita diversa*. The main characteristic of the *Helicogermslita* species is the presence of ascospores with a helical germ slit running along the entire length of the spore [69]. This phenotypic character was observed also in the isolectotype of *Rosellinia somala* stored in PAD. Unfortunately, there are no molecular data for *Helicogermslita* species in public databases and we were not able to get original material for comparison, but the isolate position of *Rosellinia somala* in the phylogram suggests that it does not belong to other Xylariaceae genera (Figure 3). The morphology of *Rosellinia somala* is similar to those of *Helicogermslita celastri* reported by Hawksworth and Lodha [69]. In the absence of molecular information, we agree with Petrini who suggested the synonymy of *Rosellinia somala* with *Helicogermslita celastri* [3].

#### 3.2.9. *Rosellinia subsimilis*

***Coniochaeta dakotensis*** Forin, Fainelli & Vizzini **nom. nov.** MycoBank MB838856 for *Rosellinia subsimilis* Sacc., Mycologia 12: 199. 1920, non *Rosellinia subsimilis* P. Karst. & Starbäck, Revue mycol., Toulouse 9 (no. 36): 160. 1887.

*Etymology:* the specific epithet refers to Dakota, the geographic area where the holotype was collected.

*Sexual stage*: *Ascomata* perithecial, gregarious, superficial, black, globose, slightly papillate, 190–250 µm diam (*n* = 10). *Asci* cylindrical without amyloid apical apparatus, (84–)90.8–*98.4*–105.9(–107) × (8–)8.1–*8.8*–9.5(–10) μm (*n* = 10), 8-spored, ascospores obliquely uniseriate. *Ascospores* asymmetrically ellipsoidal with a flattened side and rounded ends, (8–)8.7–*10.3*–12(–16.3) × (3.7–)4.1–*4.8*–5.5(–7.5) μm, Q = (1.6–)1.8–*2.2*–2.5(–2.8), Q_av_ = 2.1 (*n* = 49), brown, smooth, with a straight germ slit as long as the ascospore, one-celled.

**Figure 12:**
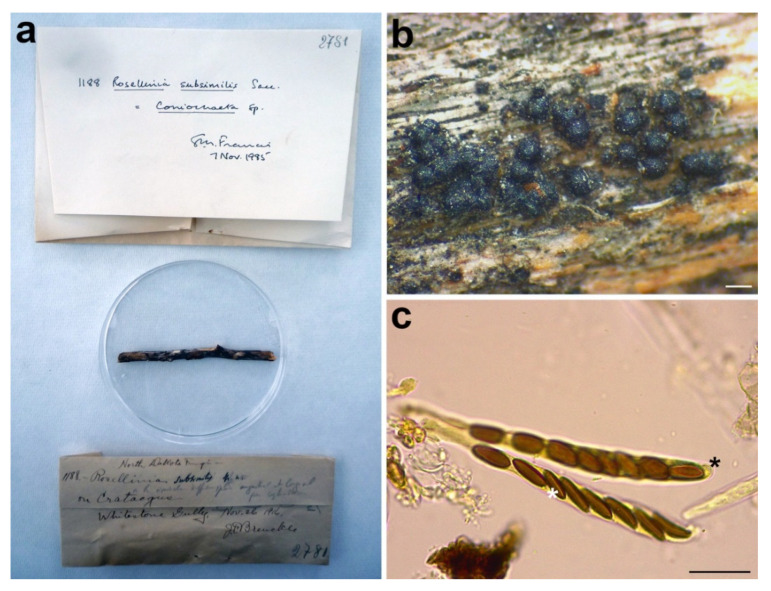
*Rosellinia subsimilis***.** (**a**) Original fungarium specimen. (**b**) Perithecia on natural substrate. (**c**) Asci without amyloid apical apparatus (black asterisk); ascospores showing germ slit (white asterisk). Bars: (**b**) = 200 μm; (**c**) = 20 μm.

*Material examined*: USA, North Dakota, Dickey Co., Whitestone Gully, on *Crataegus* sp., 26 November 1916, J. Brenckle, *n*. 1188, PAD S00035, holotype (Figure 12).

*Notes*: The holotype was morphologically revised in 1985, as reported in the label associated with the sample (Figure 12a), suggesting that the species should be placed in the genus *Coniochaeta*. Petrini treated *Rosellinia subsimilis* Sacc. (non *R. subsimilis* P. Karst. & Starbäck) among the specimens excluded from *Rosellinia*. She considered *R. subsimilis* as a *Coniochaeta* species without giving a new combination or possible synonymies [3]. The ITS sequence of *R. subsimilis* clusters with the ITS sequence of the holotype *Coniochaeta rosae* in a highly supported clade (MLB = 97%, BPP = 1). The comparison of the ITS regions reveals an identity of 97.7% between *Rosellinia subsimilis* and *Coniochaeta rosae*. Morphologically, the two species are very similar, except that the ascospores of *Rosellinia subsimilis* are smaller than those of *Coniochaeta rosae* (14–18 × 4–6 µm, *x* = 15.8 × 5.2 µm) [34]. In addition, both the species are saprobes of Rosaceae (*Coniochaeta rosae* was described on a stalk of *Rosa hissarica* in Uzbekistan) [34]. Based on the morphological and molecular analysis, *R. subsimilis* Sacc. is considered here as a new species within *Coniochaeta*. Since the name *R. subsimilis* Sacc. (1920) is invalid because it is preoccupied by *R. subsimilis* P. Karst. & Starbäck (1887), we have introduced *Coniochaeta dakotensis* as a nomen novum for the former.

## 4. Discussion

The genus *Rosellinia* has been extensively revised by Petrini considering a specific combination of phenotypic characters. She subdivided the species into seven informal morphological groups, excluding many formerly described species from the genus [2,3]. Among the types taxonomically re-evaluated, some of them come from the Saccardo fungarium stored in the PAD. In this work ITS1 and/or ITS2 sequences were obtained from nine different *Rosellinia sensu* Saccardo type specimens with the purpose of elucidating the current systematic status of these species. Coupling new morphological observations with molecular phylogenetic analyses, we introduce the new name *Coniochaeta dakotensis* (for *Rosellinia subsimilis* Sacc.) and the new nomenclatural combinations *Coniochaeta chordicola* (formerly *R. chordicola*), *C. geophila* (formerly *R. geophila*) and *Triangularia horridula* (formerly *Podospora horridula*). However, for *Rosellinia ambigua*, *R. areolata*, *R. australis*, *R. romana* and *R. somala*, we have not suggested any taxonomic change compared to the current one. An exhaustive taxonomic re-evaluation of *Rosellinia romana* and *R. somala* was not possible due to the lack of sufficient molecular information deposited in public databases. The absence of a reference database, in term of sequence data, for the species of the genus *Helicogermslita* has not allowed to have a molecular confirmation on the synonymy of *Rosellinia somala* with *H. celastri* proposed by Petrini [3]. Nevertheless, the phenotypic characters of *R. somala* are congruent with those of the species of the genus *Helicogermslita* (e.g., helicoid ascospore germ slit), in particular with *H. celastri*. This also applies to *Rosellinia romana*, which was placed in synonymy with *R. glabra* [3]; however, DNA sequences, for the latter, are not available. The morphology is an important component in fungal taxonomy and new species are continuously introduced using this approach [73]. However, molecular information enhances the value of a species description, allowing to integrate them into a modern phylogenetic context. When DNA sequence data for *Helicogersmlita* species and *Rosellinia glabra* are available, the current taxonomic position of *R. romana* and *R. somala* can be confirmed or changed. Until that moment and relying only on morphological data, we agree with the synonymies proposed by Petrini [3] for these two species. *Rosellinia mammaeformis* group, with the type of *Rosellinia romana*, is separated from the clade “*Rosellinia sensu stricto*” containing the type species *R. aquila* (Figure 3), suggesting, as also reported in other phylogenetic studies [4,6,56], that *Rosellinia sensu* Petrini is not a monophyletic clade. It is probable that a molecular study involving type specimens of the *Rosellinia aquila*, *R. emergens*, *R. mammaeformis*, *R. mammoidea* and *R. thelena* morphological groups proposed by Petrini would lead to split the genus into different genera. As well as other authors [74,75], we encourage mycologists to always generate molecular data when new species are described or when old fungal species are re-examined in order to make available useful DNA information to the entire mycological community for further studies. Once again, we demonstrate the possibility and the scientific relevance of generating molecular data from fungal type specimens stored in fungaria with the hope that, in the future, greater efforts will be employed for conducting genetic analyses on these important samples.

## Figures and Tables

**Table 1:** List and details of *Coniochaeta* and Podosporaceae specimens used in the internal transcribed spacer (ITS) phylogenetic analyses. Newly obtained sequences are reported in **bold**.

			GenBank Accession Numbers		
Original Identification	Name to Be Used	Herbarium/Strain	ITS1	ITS2	ITS
*Apiosordaria sacchari*	*Podospora sacchari*	CBS 713.70, type of *Echinopodospora sacchari* [26]	-	-	MH859915
*Apiosordaria striatispora*	*Podospora striatispora*	CBS 154.77, isotype of *Triangularia striatispora* [27]	-	-	MT784137
*Arnium arizonense*	*Triangularia arizonensis*	CBS 120289	-	-	KU955584
*Cercophora coprophila*	*Cladorrhinum coprophilum*	Z20 [28]	-	-	JN198495
*Cercophora striata*	*Triangularia striata*	SMH 4036	-	-	KX348038
*Chaetosphaeria pygmaea*	*Chaetosphaeria pygmaea*	MR 1365 [29]	-	-	AF178545
*Cladorrhinum foecundissimum*	*Cladorrhinum foecundissimum*	CBS 180.66, neotype [30]	-	-	MK926856
*Cladorrhinum hyalocarpum*	*Cladorrhinum hyalocarpum*	CBS 322.70, holotype of *Thielavia hyalocarpa* [30]	-	-	MK926857
		CBS 102198 [30]	-	-	MK926858
*Cladorrhinum intermedium*	*Cladorrhinum intermedium*	CBS 433.96, ex-holotype of *Thielavia intermedia* [30]	-	-	MK926859
*Coniochaeta acaciae*	*Coniochaeta acaciae*	MFLUCC 17-2298, ex-holotype [31]	-	-	MG062735
*Coniochaeta africana*	*Coniochaeta africana*	CBS 1200868, ex-holotype [32]	-	-	NR_137725
*Coniochaeta angustispora*	*Coniochaeta angustispora*	CBS 872.73 [26]	-	-	MH860817
*Coniochaeta arenariae*	*Coniochaeta arenariae*	MFLUCC 18-0409 [33]	-	-	MN047126
*Coniochaeta arxii*	*Coniochaeta arxii*	CBS 192.89, isotype [26]	-	-	MH862164
*Coniochaeta baysunika*	*Coniochaeta baysunika*	MFLUCC 17-0830, holotype [34]	-	-	NR_157508
*Coniochaeta boothii*	*Coniochaeta boothii*	CBS 381.74, type of *Thielavia boothii* [26]	-	-	NR_159776
*Coniochaeta canina*	*Coniochaeta canina*	CBS 133243, ex-holotype of *Lecythophora canina* [35]	-	-	NR_120211
*Coniochaeta cateniformis*	*Coniochaeta cateniformis*	CBS 131709, holotype of *Lecythophora cateniformis* [26]	-	-	MH865902
*Coniochaeta cephalothecoides*	*Coniochaeta cephalothecoides*	L821 [36]	-	-	KY064029
*Coniochaeta cipronana*	*Coniochaeta cipronana*	CBS 144016, holotype [37]	-	-	NR_157478
*Coniochaeta coluteae*	*Coniochaeta coluteae*	MFLUCC 17-2299, ex-holotype [31]	-	-	MG137251
*Coniochaeta cymbiformispora*	*Coniochaeta cymbiformispora*	NBRC 32199, ex-holotype	-	-	LC146726
*Coniochaeta cypraeaespora*	*Coniochaeta cypraeaespora*	CBS 365.93, type [26]	-	-	MH862420
*Coniochaeta decumbens*	*Coniochaeta decumbens*	CBS 153.42, holotype of *Margarinomyces decumbens* [38]	-	-	NR_144912
*Coniochaeta discoidea*	*Coniochaeta discoidea*	CBS 158.80, type of *Poroconiochaeta discoidea* [26]	-	-	NR_159779
*Coniochaeta discospora*	*Coniochaeta discospora*	CBS 168.58, type of *Sordaria discospora* [26]	-	-	MH857740
*Coniochaeta ellipsoidea*	*Coniochaeta ellipsoidea*	CBS 137.68, type [26]	-	-	MH859091
*Coniochaeta endophytica*	*Coniochaeta endophytica*	AEA 9094, type [39]	-	-	EF420005
*Coniochaeta euphorbiae*	*Coniochaeta euphorbiae*	CBS 139768, ex-type [40]	-	-	KP941076
*Coniochaeta extramundana*	*Coniochaeta extramundana*	CBS 247.77, type [26]	-	-	MH861057
*Coniochaeta fasciculata*	*Coniochaeta fasciculata*	CBS 205.38, holotype of *Margarinomyces fasciculatus* [38]	-	-	NR_154770
*Coniochaeta fodinicola*	*Coniochaeta fodinicola*	CBS 136963, holotype [41]	-	-	JQ904603
*Coniochaeta gigantospora*	*Coniochaeta gigantospora*	ILLS 60816, type [35]	-	-	NR_121521
*Coniochaeta hoffmannii*	*Coniochaeta hoffmannii*	CBS 245.38, holotype of *Margarinomyces hoffmannii* [42]	-	-	NR_167688
*Coniochaeta iranica*	*Coniochaeta iranica*	CBS 139767 [40]	-	-	KP941078
*Coniochaeta ligniaria*	*Coniochaeta ligniaria*	CBS 424.65 [26]	-	-	MH858650
*Coniochaeta lignicola*	*Coniochaeta lignicola*	CBS 267.33, lectotype of *Lecythophora lignicola* [35]	-	-	NR_111520
*Coniochaeta luteorubra*	*Coniochaeta luteorubra*	CBS 131710, holotype *Lecythophora luteorubra* [26]	-	-	MH865901
*Coniochaeta luteoviridis*	*Coniochaeta luteoviridis*	CBS 206.38, holotype of *Margarinomyces luteoviridis* [38]	-	-	NR_154769
*Coniochaeta marina*	*Coniochaeta marina*	MFLUCC 18-0408, ex-holotype [43]	-	-	MK458764
*Coniochaeta mutabilis*	*Coniochaeta mutabilis*	CBS 157.44, holotype of *Margarinomyces mutabilis* [35]	-	-	NR_111519
*Coniochaeta navarrae*	*Coniochaeta navarrae*	CBS 141016, ex-holotype [44]	-	-	NR_154808
*Coniochaeta nepalica*	*Coniochaeta nepalica*	NBRC 30584, ex-type	-	-	LC146727
*Coniochaeta ostrea*	*Coniochaeta ostrea*	CBS 507.70, type of *Coniochaetidium ostreum* [26]	-	-	NR_159772
*Coniochaeta polymorpha*	*Coniochaeta polymorpha*	CBS 132722, holotype [35]	-	-	NR_121473
*Coniochaeta polysperma*	*Coniochaeta polysperma*	CBS 669.77, isotype [26]	-	-	MH861109
*Coniochaeta prunicola*	*Coniochaeta prunicola*	CBS 120875, holotype [32]	-	-	NR_137037
*Coniochaeta punctulata*	*Coniochaeta punctulata*	CBS 159.80, isotype of *Poroconiochaeta punctulata* [26]	-	-	MH861254
*Coniochaeta rosae*	*Coniochaeta rosae*	MFLUCC 17-0810, holotype [34]	-	-	NR_157509
*Coniochaeta savoryi*	*Coniochaeta savoryi*	CBS 725.74, isotype of *Thielavia savoryi* [26]	-	-	MH860890
*Coniochaeta simbalensis*	*Coniochaeta simbalensis*	NFCCI 4236, ex-holotype [45]	-	-	NR_164024
*Coniochaeta taeniospora*	*Coniochaeta taeniospora*	CBS 141014, ex-epitype [44]	-	-	KU762324
*Coniochaeta tetraspora*	*Coniochaeta tetraspora*	CBS 139.68 [26]	-	-	MH859093
*Coniochaeta velutina*	*Coniochaeta velutina*	CBS 120874 [32]	-	-	GQ154542
*Coniochaeta verticillata*	*Coniochaeta verticillata*	CBS 816.71, holotype of *Ephemeroascus verticillatus* [26]	-	-	NR_159774
*Coniochaeta vineae*	*Coniochaeta vineae*	KUMCC 17-0322, ex-holotype [46]	-	-	MN473469
*Lasiosphaeria ovina*	*Lasiosphaeria ovina*	SMH3286 [47]	-	-	AY587931
*Podospora bulbillosa*	*Podospora bulbillosa*	CBS 304.90, ex-holotype of *Cladorrhinum bulbillosum* [30]	-	-	MK926861
*Podospora fimicola*	*Podospora fimicola*	CBS 482.64, ex-epitype of *Schizothecium fimicola* [30]	-	-	MK926862
*Podospora setosa*	*Triangularia setosa*	FMR 12787 [27]	-	-	MT784144
***Rosellinia ambigua***	*Coniochaeta ambigua*	**PAD** **S00027: fungarium Saccardo, holotype**	**MW626895**	**MW626903**	-
***Rosellinia chordicola***	*Coniochaeta chordicola* (Sacc.) Forin, Fainelli & Vizzini	**PAD S00030: fungarium Saccardo, holotype**	**MW626898**	**MW626905**	-
***Rosellinia geophila***	*Coniochaeta geophila* (E. Bommer, M. Rousseau & Sacc.) Forin, Fainelli & Vizzini	**PAD S00031: fungarium Saccardo, holotype**	-	**MW626906**	-
***Rosellinia horridula***	*Triangularia horridula* (Sacc.) Forin, Fainelli & Vizzini	**PAD S00032: fungarium Saccardo, holotype**	**MW626899**	**MW626907**	-
***Rosellinia subsimilis***	*Coniochaeta dakotensis* Forin, Fainelli & Vizzini	**PAD S00035: fungarium Saccardo, holotype**	**MW626902**	**MW626910**	-
*Triangularia allahabadensis*	*Triangularia allahabadensis*	CBS 724.68, ex-holotype of *Sordaria allahabadensis* [30]	-	-	MK926865
*Triangularia anserina*	*Triangularia anserina*	CBS 433.50 [30]	-	-	MK926864
*Triangularia backusii*	*Triangularia backusii*	CBS 539.89, isotype [30]	-	-	MK926866
		CBS 106.77 [30]	-	-	MK926867
*Triangularia bambusae*	*Triangularia bambusae*	CBS 352.33, type of *Trigonia bambusae* [30]	-	-	MK926868
*Triangularia batistae*	*Triangularia batistae*	CBS 381.68, type [27]	-	-	MT784140
*Triangularia longicaudata*	*Triangularia longicaudata*	CBS 252.57, type [30]	-	-	MK926869
		FMR 12782 [27]	-	-	MT784142
*Triangularia pauciseta*	*Triangularia pauciseta*	CBS 451.62 [30]	-	-	MK926870
*Triangularia phialophoroides*	*Triangularia phialophoroides*	CBS 301.90, ex-holotype of *Cladorrhinum phialophoroides* [30]	-	-	MK926871
*Triangularia setosa*	*Triangularia setosa*	CBS 311.58 [30]	-	-	MK926872
*Triangularia verruculosa*	*Triangularia verruculosa*	CBS 148.77 [30]	-	-	MK926874
*Zopfiella tetraspora*	*Triangularia tetraspora*	CBS 245.71, type of *Podospora buffonii* [26]	-	-	MH860097
		IFO 32904	-	-	AY999130

**Table 2:** List and details of Xylariaceae and Hypoxylaceae specimens used in the combined ITS-LSU-TUB2 phylogenetic analysis. Newly obtained sequences are reported in **bold**.

			GenBank Accession Numbers				
Original Identification	Name to Be Used	Herbarium/Strain	ITS1	ITS2	ITS	LSU	TUB2
*Amphirosellinia fushanensis*	*Amphirosellinia fushanensis*	HAST 91111209, holotype [48]	-	-	GU339496	-	GQ495950
*Amphirosellinia nigrospora*	*Amphirosellinia nigrospora*	HAST 91092308, holotype [48]	-	-	GU322457	-	GQ495951
*Annulohypoxylon annulatum*	*Annulohypoxylon annulatum*	CBS 140775, ex-epitype [6]	-	-	KY610418	KY610418	-
*Annulohypoxylon areolatum*	*Annulohypoxylon areolatum*	MFLUCC 14-1233, EK14019 ex-epitype [49]	-	-	KX376327	-	KX376344
*Annulohypoxylon bovei* var. *microspora*	*Annulohypoxylon bovei* var. *microspora*	YMJ 90081914 [48,50]	-	-	EF026141	-	AY951654
*Annulohypoxylon truncatum*	*Annulohypoxylon truncatum*	CBS 140778, ex-epitype [6,49]	-	-	KY610419	KY610419	KX376352
*Coniolariella ershadii*	*Coniolariella ershadii*	Co48, CBS 119785, ex-type [51]	-	-	GU553328	GU553331	-
*Coniolariella gamsii*	*Coniolariella gamsii*	Co27, CBS 114379, ex-type [51]	-	-	GU553325	GU553329	-
*Coniolariella hispanica*	*Coniolariella hispanica*	Co125, CBS 124506, ex-type [51]	-	-	GU553323	GU553353	-
*Coniolariella macrothecia*	*Coniolariella macrothecia*	Co127, CBS 125772, ex-type [51]	-	-	GU553324	GU553354	-
*Creosphaeria sassafras*	*Creosphaeria sassafras*	STMA 14087 [6]	-	-	KY610411	KY610468	KX271258
*Dematophora bunodes*	*Dematophora bunodes*	CBS 123584 [4]	-	-	MN984617	-	MN987243
		CBS 123597 [4]	-	-	MN984618	-	MN987244
*Dematophora pepo*	*Dematophora pepo*	CBS 123592 [4]	-	-	MN984620	-	MN987246
*Hypoxylon fragiforme*	*Hypoxylon fragiforme*	MUCL51264, ex-epitype [52]	-	-	KM186294	KM186295	KM186293
*Hypoxylon rickii*	*Hypoxylon rickii*	MUCL 53309, ex-epitype [53]	-	-	KC968932	-	KC977288
*Nemania abortiva*	*Nemania abortiva*	BiSH 467, holotype [48]	-	-	GU292816	-	GQ470219
*Nemania bipapillata*	*Nemania bipapillata*	HAST 90080610 [48]	-	-	GU292818	-	GQ470221
*Nemania primolutae*	*Nemania primolutae*	HAST 91102001 [48,54]	-	-	EF026121	-	EF025607
*Rosellinia abscondita*	*Rosellinia abscondita*	CBS 447.89 [55]	-	-	FJ175180	KF719208	-
*Rosellinia aquila*	*Rosellinia aquila*	MUCL 51703 [6]	-	-	KY610392	KY610460	KX271253
***Rosellinia areolata***	*Annulohypoxylon areolatum*	**PAD S00028: fungarium Saccardo, holotype**	**MW626896**	**-**	-	-	-
***Rosellinia australis***	*Coniolariella limoniispora*	**PAD S00029: fungarium Saccardo, holotype**	**MW626897**	**MW626904**	-	-	-
		AH24323 [56,57]	-	-	AY908997	EF489464	-
*Rosellinia australiensis*	*Rosellinia australiensis*	CBS 142160, ex-holotype [58]	-	-	KY979742	KY979797	-
*Rosellinia buxi*	*Dematophora buxi*	JDR 99 [48]	-	-	GU300070	-	GQ470228
*Rosellinia corticum*	*Rosellinia corticum*	MUCL 51693 [6]	-	-	KY610393	KY610461	KX271254
*Rosellinia limonispora*	*Coniolariella limoniispora*	CBS 382.86	-	-	KF719199	KF719211	-
*Rosellinia mammiformis*	*Rosellinia mammiformis*	CBS 445.89	-	-	KF719200	KF719212	-
*Rosellinia merrillii*	*Rosellinia merrillii*	HAST 89112601 [48]	-	-	GU300071	-	GQ470229
*Rosellinia necatrix*	*Dematophora necatrix*	CBS 349.36 [56]	-	-	AY909001	KF719204	KY624310
		W97	-	-	DF977487	DF977487	DF977466
*Rosellinia nectrioides*	*Rosellinia nectrioides*	CBS 449.89 [4]	-	-	MN984622	MN984628	-
***Rosellinia romana***	*Rosellinia glabra*	**PAD S00033: fungarium Saccardo, holotype**	**MW626900**	**MW626908**	-	-	-
***Rosellinia somala***	*Helicogermslita celastri*	**PAD S00034: fungarium Saccardo, isolectotype**	**MW626901**	**MW626909**	-	-	-
*Xylaria bambusicola*	*Xylaria bambusicola*	WPS 205, holotype [48,50]	-	-	EF026123	-	AY951762
*Xylaria hypoxylon*	*Xylaria hypoxylon*	CBS 122620 [6,59]	-	-	KY204024	KY610495	KX271279

## Data Availability

New sequence data are available in NCBI GenBank and the accession numbers are reported in Table 1 and Table 2_._ The Illumina sequencing data are not publicly available because they contain data not involved in this study but currently investigated for other purposes. These data are available upon request to corresponding authors (niccolo.forin@unipd.it, barbara.baldan@unipd.it).
